# Developing a concise multivariable predictive model for cesarean delivery following neuraxial analgesia during labor: a prospective observational cohort study

**DOI:** 10.1016/j.bjane.2026.844732

**Published:** 2026-01-25

**Authors:** Paula Daniele Lopes da Costa, Murilo Henrique da Veiga Ferreira, Joelcio Francisco Abbade, Claudia Garcia Magalhães, Norma Sueli Pinheiro Módolo, Guilherme Antonio Moreira de Barros, Gabriel Ricardo Correa Turco, Pedro Henrique Esteves Trindade, Paulo do Nascimento Junior

**Affiliations:** a Universidade do Estado de São Paulo (UNESP), Faculdade de Medicina de Botucatu, Department of Surgical Specialties and Anesthesiology, São Paulo, SP, Brazil; b Universidade do Estado de São Paulo (UNESP), Faculdade de Medicina de Botucatu, Department of Gynecology and Obstetrics, São Paulo, SP, Brazil; c Michigan State University (MSU), College of Veterinary Medicine, Department of Large Animal Clinical Sciences, East Lansing, United States

**Keywords:** Cesarean section, Epidural analgesia, Labor pain, Obstetric analgesia, Predictive values of tests

## Abstract

**Background:**

Sometimes, planned vaginal deliveries with neuraxial analgesia may result in unplanned cesareans. We aimed to determine the incidence of cesarean among parturients receiving neuraxial analgesia for vaginal delivery, identify associated factors, and develop a predictive model.

**Methods:**

In this prospective observational cohort study, we evaluated parturients receiving neuraxial analgesia for vaginal delivery and analyzed factors associated with progression to cesarean. Multiple logistic regression with a *step-up* procedure was performed. The dataset was split into training (70%) and testing (30%) databases, with the latter used to assess performance metrics. Bootstrap validation with 5,000 repetitions was performed.

**Results:**

We evaluated 331 parturients and 94 (28.4%) underwent cesarean. Variables differing between cesarean and vaginal delivery groups (p < 0.05) included patient age, body mass index, gestational age, cervical dilation at analgesia initiation, time under analgesia, labor conducted/monitored by nurses, and oxytocin use after analgesia initiation. Three variables remained predictive [odds ratio (95% Confidence Interval (95% CI))]: patient age: 1.0436 (1.0091 to 1.0835), p = 0.018; time under analgesia: 1.0043 (1.0008 to 1.0081), p = 0.018; and oxytocin use after analgesia initiation: 0.0921 (0.0400 to 0.1945), p < 0.001. Predictive area under the curve (95% CI) was 71.8% (60.5%‒83.1%). Arrest of descent (35.1%) and fetal distress (34.0%) were the leading indications for cesarean.

**Conclusions:**

Among parturients receiving neuraxial labor analgesia, older patients, longer analgesia duration, and no oxytocin use after analgesia initiation increase the probability of cesarean, with moderate predictivity. Arrest of descent and fetal distress were the main causes of cesarean.

## Introduction

During labor, neuraxial injection of local anesthetics may provide relaxation of the muscles of the pelvic floor and abdominal wall, which are important components in the process of fetal rotation and correct cephalic positioning, prolonging the second stage of labor, increasing the risk of instrumental vaginal delivery, in addition to increasing the use of oxytocin to optimize uterine contractility and avoid dystocia.^1^ Although neuraxial analgesia is associated with these outcomes, studies on the increased risk of cesarean delivery have conflicting results.^2^^,^^3^ Some factors, such as maternal age and the use of oxytocin during labor, have been associated with variations in the incidence of cesarean delivery.^4^^,^^5^ Other elements, including the time of day when labor occurs and the type of healthcare provider, i.e., nurses, midwives or obstetricians,^6^^,^^7^ have also been investigated as potential contributors to cesarean rates. A predictive model for cesarean delivery in nulliparous patients during hospital admission revealed a 71% accuracy, and that the factors associated were advanced maternal age, shorter maternal height, greater gestational age, labor lasting more than 24 hours, irregular contractions, less cervical dilation, and higher fetal station.^8^ Another predictive model evaluated the risk of cesarean delivery after induction of labor and found that nulliparity and macrosomia were the factors with the highest odds ratios.^9^

However, during labor analgesia, the extent to which some of these factors influence cesarean delivery, particularly when considered in combination, remains to be fully elucidated. We hypothesized that a limited set of clinical variables, easy to collect and observe, selected at the time of neuraxial analgesia, could predict subsequent cesarean delivery. Given the uncertainties about the risk of cesarean during neuraxial labor analgesia for vaginal delivery, this study aimed to determine the incidence of cesarean among parturients who received neuraxial labor analgesia, to identify associated maternal and gestational factors and, thus, construct a predictive model. The causes of cesarean were also evaluated in these patients.

## Methods

This is a prospective observational cohort study, approved by the Institutional Ethics Committee, performed with parturients of the Maternity at the Hospital das Clínicas of Botucatu Medical School, Brazil, conducted in accordance with the recommendations of the Strengthening the Reporting of Observational Studies in Epidemiology (STROBE) and the Transparent Reporting of a multivariable prediction model for Individual Prognosis Or Diagnosis (TRIPOD statement).

During the period between March 2022 and June 2023, all consecutive patients with indication for labor analgesia were invited to participate in the study after signing the informed consent form. Patients of any age who received neuraxial analgesia (spinal, epidural, or combined spinal-epidural) for vaginal delivery were included. In the case of fetal demise, parturients did not participate in the study. The obstetricians requested the analgesic procedure according to their clinical evaluation of pain intensity and cervical dilation, on an individual basis.

The index time for collecting the variables was the onset of analgesia. The variables chosen for the analysis of the possible association with cesarean obtained at the index time were patient age (years); weight (kg); height (cm); body mass index (kg.m^-^²); gestational age (weeks and days); physical status according to American Society of Anesthesiologists (classification, I to VI); cervical dilation at the time of analgesia administration (cm); previous pregnancies (number of previous pregnancies); previous vaginal deliveries (number and percentage); pain score before analgesia (scale from zero (no pain) to 10 (maximum possible pain)); analgesia performed at night (after 8 pm, number of patients and percentage); analgesia technique (spinal, epidural or combined spinal/epidural, number and percentage); use of oxytocin before analgesia initiation (number of patients and percentage); and labor monitored/accompanied by nursing (number of patients and percentage). The variables chosen for the analysis of the possible association with cesarean obtained after the onset (post-index) of analgesia were time under analgesia (minutes); and use of oxytocin after analgesia initiation (number of patients and percentage).

The choice of the neuraxial technique, whether spinal, epidural, or combined (spinal-epidural), with or without the use of an epidural catheter, as well as the selected drugs and their respective doses, was at the discretion of the attending anesthesiologist.

In cases where cesarean delivery was indicated by the obstetrician, or according to the patient’s will, we recorded the reasons for the indication, and the attending anesthesiologist decided on the anesthetic technique for the cesarean.

The primary outcome of the study was to evaluate the percentage of parturients who received neuraxial analgesia and underwent an unplanned cesarean delivery, to identify the possible associated factors, and to construct a predictive model. The secondary outcome was the identification of the causes of cesarean delivery.

### Statistical analysis

Based on the number of labor analgesia procedures performed in 2021 (204 cases), according to local records, we made a preliminary convenience analysis with 194 consecutive cases and found that four independent variables were associated with cesarean delivery: body mass index; longer gestational age; longer time under labor analgesia; and no oxytocin use after analgesia. Using the sample size equation for observational studies involving logistic regression as suggested by Bujang et al. (n = 100 +50*i*, where *i* refers to the number of independent variables in the model),^10^ the minimum number required for the analysis would be 300 patients. We included approximately 10% more participants than the minimum required sample size, aiming to compensate for potential data loss due to incomplete forms or missing critical information that could compromise the quality and completeness of the dataset, resulting in a total of 331 patients.

To choose the set of variables to compose the predictive model, the difference between groups (vaginal delivery vs. cesarean section) was investigated. Quantitative variables were analyzed via two-tailed Student’s *t-*test (stats::t.test) when a Gaussian distribution was verified via the Cramer-Von Mises test (nortest::cvm.test) or the two-tailed Mann-Whitney test (stats::wilcox.test) when the variables did not meet this assumption. A chi-square test (stats::chisq) was used for the qualitative variables.

Following, the dataset was divided into training and testing databases. The training database contained 70% of the randomly selected patients, while the testing database had 30% of the remaining patients using a random stratification sampling by groups without replacements (vaginal delivery and cesarean). The proportion of events (cesareans and vaginal deliveries) was maintained in both databases, according to that observed in the dataset. Then, the variables that were significantly different between the groups were used as predictive variables and the groups were used as predictor variables in a multiple binomial logistic regression model using the training database (stats::glm) (full model). The model was subjected to a *step-up* procedure in which non-significant predictive variables were manually removed from the model. This process was repeated sequentially until all predictive variables were significant (final model). The best model was chosen according to the Root Mean Square Error (RMSE), Akaike Information Criterion (AIC), Bayesian Information Criterion (BIC) and the Area Under the Curve (AUC) (jtools::summ; sjstats::rmse; lmtest::lrtest; pROC::roc; and pROC::ci.auc). The AUC between models was compared via the DeLong test (pROC::roc.test) and we also used a null model for comparisons. The Hosmer-Lemeshow goodness-of-fit test (glmtoolbox:: hltest) was performed to evaluate the final model. Variance Inflation Factor (VIF) was used to assess the multicollinearity among predictive variables (car::vif). A bootstrap resampling with 5,000 repetitions was performed to internally validate the linear and slope coefficients of the final model (car::boot). Least Absolute Shrinkage and Selection Operator (LASSO) with 10-fold cross-validation was applied to the final predictive model obtained after the *step-up* procedure using the training database (glmnet::cv.glmnet and glmnet::glmnet). Events per variable were calculated using the final Bayesian logistic regression model (4 chains, 2,000 iterations, 1,000 warmup) with training database. Expected posterior variance was estimated via posterior predictive simulations: multiple datasets were simulated from the fitted model, refitted, and posterior variances of coefficients averaged to quantify the expected uncertainty of each parameter (brm::brm).

The purpose of the final model was to present a set of variables that could predict the occurrence of cesarean delivery. Finally, the testing database was used to evaluate the quality of the model and to develop the AUC and its 95% Confidence Interval, obtained from the Receiver Operating Characteristics (ROC) curve with 1,001 replicates by bootstrap (pROC::roc; pROC::ci.auc; and pROC::ci.coords). A calibration plot was constructed with the testing database, containing 30% of patients and maintaining the proportion of cesareans observed in the dataset. Decision curve analysis was performed using threshold probabilities ranging from 0.05 to 0.80, in increments of 0.005, and the 95% Confidence Interval was calculated by a bootstrap with 100 repetitions (rmda:: decision_curve and rmda::plot_decision_curve); p-values < 0.05 were considered significant. Statistical analyses were conducted using *R* programming language in the RStudio software (Version 4.1.0; 2021–06–29; RStudio, Inc.). Functions and packages were presented as ‘package::function’, corresponding to computational language in *R*.

## Results

**Figure 1:**
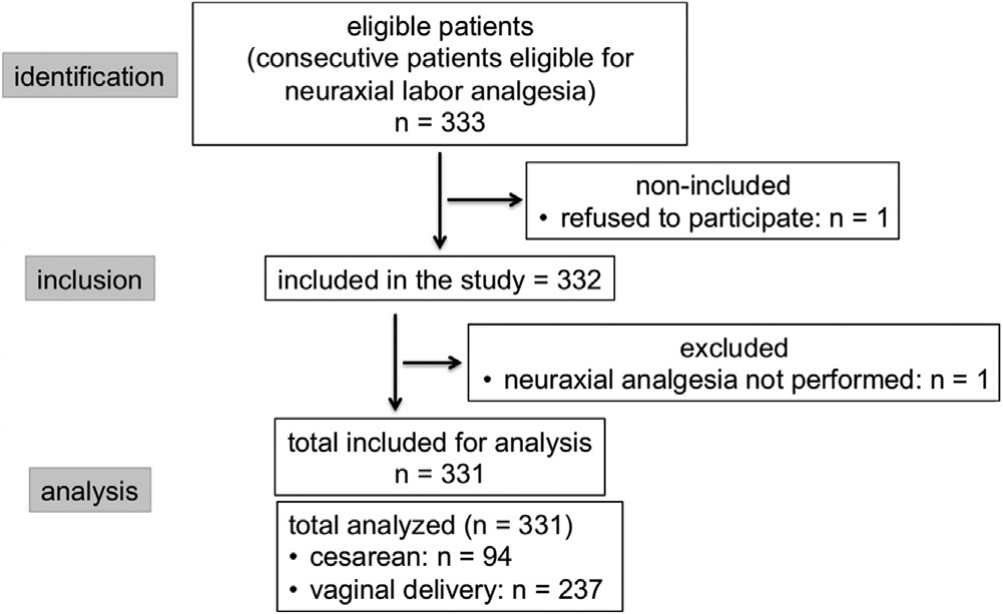
Flowchart.

During the study period, 331 patients received neuraxial analgesia, and 94 (28.4%) progressed to cesarean delivery (Fig. 1). The quantitative and qualitative variables analyzed are presented in Table 1, in relation to the occurrence of vaginal and cesarean deliveries, with their statistical values according to univariate analysis.

**Table 1:** Distribution of quantitative and qualitative variables between parturients who underwent vaginal or cesarean delivery after neuraxial labor analgesia.

Variables	Vaginal Delivery (n = 237)	Cesarean (n = 94)	p-value
Age (years)	24 (20 – 29)	25 (21 – 29)	0.032
Weight (kg)	75 (68 – 87)	78 (69 – 91)	0.144
Height (cm)	1.63 (1.58 – 1.67)	1.62 (1.57 – 1.67)	0.289
Body mass index (kg.m^-^²)	28.7 (25.8 – 33.2)	30.4 (27.6 – 34.5)	0.018
Gestational age [weeks (w)/days (d)]	39w/1d (38w/1d – 40w/1d)	39w/6d (38w/4d – 40w/3d)	0.006
Physical status			0.308
ASA^a^ 2	204 (86.1%)	76 (80.8%)	
ASA 3	33 (13.9%)	18 (19.1%)	
Cervical dilation (cm)^b^	7 (7 – 8)	7 (6 – 8)	0.028
Previous pregnancies	1 (1 – 2)	1 (1 – 2)	0.422
Previous vaginal deliveries	63 (26.6%)	21 (22.3%)	0.424
Time under analgesia (min)	103 (55 – 184)	162 (102 – 233)	< 0.001
Numerical pain scale^c^ (before analgesia)	10 (10 – 10)	10 (10 – 10)	0.751
Analgesia performed at night (after 8 pm)	101 (42.7%)	38 (40.4%)	0.787
Analgesia technique			0.397
Spinal^d^	181 (76.3%)	66 (70.2%)	
Spinal + epidural^e^	38 (16.0%)	21 (22.3%)	
Epidural^f^	18 (7.6%)	7 (7.4%)	
Use of oxytocin before analgesia	54 (22.7%)	12 (12.7%)	0.057
Use of oxytocin after analgesia	155 (65.6%)	22 (23.4%)	< 0.001
Labor monitored/accompanied by nursing	23 (9.7%)	22 (23.4%)	0.002

Data are presented as the median (1^st^ – 3^rd^ quartiles) or number of patients (%).

a American Society of Anesthesiologists.

b Dilation of the uterine cervix at the time of analgesia administration.

c Scale from zero (no pain) to 10 (maximum possible pain).

d Drugs and doses in groups Vaginal delivery and Cesarean, respectively (mean ± Standard Deviation [SD]): hyperbaric bupivacaine (mg), 2.6 ± 0.6 and 2.8 ± 1.0, and sufentanil (μg), 4.9 ± 0.7 and 5.0 ± 0.8.

e Drugs and doses in groups Vaginal delivery and Cesarean, respectively (mean ± SD): hyperbaric bupivacaine (mg), 2.7 ± 0.9 and 2.6 ± 0.3, sufentanil (μg), 5.5 ± 2.3 and 5.0 ± 1.3, and ropivacaine (mg), 18.2 ± 8.6 and 19.8 ± 15.0.

f Drugs and doses in groups Vaginal delivery and Cesarean, respectively (mean ± SD): ropivacaine (mg), 27.7 ± 11.4 and 28.3 ± 14.6.

Following the split, the training database (70%, 232 out of 331 patients) was composed by 166 (71.5%) cases of vaginal delivery and 66 (28.5%) of cesarean, while the testing database (30%, 99 out of 331 patients) was composed by 71 (71.7%) cases of vaginal delivery and 28 (28.3%) of cesarean.

Table 2 shows the fit parameters of each model, before and after the *step-up* procedure. A null model is also presented for comparison. Unlike the full model, with all variables initially identified as significant, the final model presented all slope coefficients as significant. Their AUC was considered equivalent (p = 0.413).

**Table 2:** Findings of the fit parameters for each model before and after the *step-up* procedure using the training database. A null model is presented for comparison.

Parameters	Null Model	Full Model (7 identified variables)	Final Model (3 selected variables)
		Before the *step-up*	After *step-up*
χ^2^ (df); p-value^a^	0.00 (0); p = not applicable	63.3 (7); p < 0.001	55.7 (3); p < 0.001
*AIC*^b^	279.1 (-268.0 to 826.1)	205.1 (-196.8 to 607.1)	204.6 (-196.5 to 605.8)
*BIC*^c^	282.5 (-271.2 to 836.3)	231.8 (-222.5 to 686.1)	218.0 (-209.2 to 645.3)
*RMSE*^d^	0.4512 (-0.4331 to 1.3355)	0.3796 (-0.3645 to 1.1236)	0.3912 (-0.3755 to 1.1579)
AUC^e^ (95% confidence interval)	50.0 (50.0 to 50.0)^b^	83.0 (76.6 to 89.4)^c^	81.5 (75.5 to 87.5)^d^
Slope coefficients (n)	0	7	3
Significant slope coefficients (n)	0	3	3

a χ² is the Chi-Square test, and df refers to degrees of freedom. AIC, Akaike Information Criterion; BIC, Bayesian Information Criterion; RMSE, Root Mean Square Error; AUC, Area Under the Curve.

b p < 0.0001, DeLong test comparing the AUC between null and final models.

c p = 0.0001, DeLong test comparing the AUC between null and full models.

d p = 0.413, DeLong test comparing the AUC between full and final models. The training database contains 70% of the randomly selected patients using a random stratification sampling by groups (232 patients: 166 [71.5%] cases of vaginal delivery and 66 [28.5%]) of cesarean).

In the final model, no evidence of lack of fit between predicted probabilities and observed outcomes were supported by the Hosmer-Lemeshow goodness-of-fit test (statistic = 7.4; degrees of freedom = 8; p = 0.487). Collinearity issues were not identified based on the VIFs of patient age (1.02), time under neuraxial analgesia (1.07), and oxytocin use after analgesia (1.07). Table 3 presents outcomes from the bootstrap resampling approach. The older the patients were (β = 0.043; p = 0.018) the greater the probability of cesarean (1.0436 increase for each one-year increase), and the longer the time under analgesia (β = 0.004; p = 0.018) the greater the probability of cesarean (1.0043 increase for each one-minute increase), given the positive slope coefficients (β) and significance (p) of these variables. On the other hand, the use of oxytocin after analgesia (β = -2.386; p < 0.001) decreased the probability of cesarean, given the negative slope coefficient (β) and significance (p) of this variable. The odds ratio indicates that the administration of oxytocin after delivery of analgesia is a factor that decreases the probability of occurrence of cesarean, while the greater age of patients and the longer time under analgesia are factors that increase the probability of occurrence of cesarean (Table 4).

**Table 3:** Findings of the bootstrap resampling approach on the predictive final model using the training database.

Parameters	Original estimated	p-value	Bootstrap		
			Bias	Standard error	95% CI
Linear coefficient (α)	-12.289		-0.995	5.642	-23.802 to -2.6488
Slope coefficients (β)					
Patient age	0.043	0.018	0.003	0.020	0.008 to 0.084
Time under neuraxial analgesia	0.004	0.018	0.001	0.002	0.001 to 0.009
Oxytocin use after analgesia	-2.386	< 0.001	-0.090	0.448	-3.185 to -1.515

The training database contains 70% of the randomly selected patients using a random stratification sampling by groups (232 patients: 166 [71.5%] cases of vaginal delivery and 66 [28.5%] of cesarean).

**Table 4:** Findings of the predictive final model after the *step-up* procedure using the training database.

Parameters	Estimated value	Standard error	*Z* value	p-value	Odds ratio (95% CI)
Linear coefficient (α)	-12.289	4.978	-2.469	0.014	0.0001 (0.0001 to 0.0463)
Slope coefficients (β)					
Patient age	0.043	0.018	2.359	0.018	1.0436 (1.0091 to 1.0835)
Time under neuraxial analgesia	0.004	0.001	2.367	0.018	1.0043 (1.0008 to 1.0081)
Oxytocin use after analgesia	-2.386	0.401	-5.951	< 0.001	0.0921 (0.0400 to 0.1945)

The training base contains 70% of the randomly selected patients using a random stratification sampling by groups (232 patients: 166 [71.5%] cases of vaginal delivery and 66 [28.5%] of cesarean).

Table 5 shows the values of sensitivity, specificity, and AUC of the ROC curve of the *step-up* model using the testing and training databases that include patient age, time under labor analgesia, and use of oxytocin after the administration of labor analgesia. The values of the testing database indicate the performance metrics of the predictive model.

**Table 5:** Outcomes of the Receiver Operating Characteristic (ROC) curve of the training database and of the predictive final model using the testing database.

Parameters	Values (95% CI)	
	Training database	Testing database
**Optimal cutoff point (%)**	37.9 (16.2 to 54.4)	25.5 (8.5 to 62.9)
**Specificity (%)**	73.8 (55.5 to 91.5)	65.2 (36.2 to 100.0)
**Sensibility (%)**	81.8 (60.1 to 95.5)	78.6 (35.7 to 100.0)
**Area under the curve (%)**	81.5 (75.5 to 87.5)	71.8 (60.5 to 83.1)

The training database contains 70% of the randomly selected patients using a random stratification sampling by groups (232 patients: 166 [71.5%] cases of vaginal delivery and 66 [28.5%] of cesarean), while testing database has 30% of the reminiscent patients (99 patients: 71 [71.7%] cases of vaginal delivery and 28 [28.3%] of cesarean).

The penalized regression (LASSO) shows the same ranking of importance of the variables included in the final predictive model obtained through the *step-up* procedure using the training database (Supplementary Material Table 1S).

Expected posterior variance for the slope coefficients of all three parameters was very small, suggesting precision of the parameters in estimating their respective effects in the model, while the linear coefficient was relatively large, reflecting a moderate degree of imprecision in its estimation (Supplementary Material Table 2S).

Calibration on the testing database is suboptimal, especially at lower predicted probabilities (Supplementary Material Fig. 1S,), potentially due to the limited sample size and model overfitting. Using the optimal cutoff point determined from the training database, we identified 52 true negative cases, 16 true positives, 13 false positives, and 18 false negatives in the testing database. In the decision curve analysis (Supplementary Material Fig. 2S,), the final predictive model demonstrated a higher net benefit across a wide range of threshold probabilities compared to the strategies of treating all or treating none. This indicates that the model offers potential clinical utility, particularly within threshold probabilities range of approximately 0.15 to 0.45, for identifying parturients who may require cesarean delivery.

The equation for estimating the probability of an unplanned cesarean in patients under labor analgesia for vaginal delivery is described below, using the fixed effects of the model: $P=(\frac{1}{1+e^{-(-12.289+Age*0.043+Analgesia*0.004+Oxytocin*-2.386)}})*100$; p is the probability of cesarean; *e* is the Euler number ∼ 2.718281828459045235360287; Age is the patient age (years); Analgesia is the time under neuraxial analgesia (minutes); Oxytocin is oxytocin use after analgesia (0 = no, 1 = yes).

The indications for cesarean were arrest of fetal descent (n = 33; 35.1%), fetal distress (n = 32; 34.0%), arrest of cervical dilation (n = 21; 22.3%), and patient’s decision to withdraw from vaginal delivery (n = 8; 8.5%).

## Discussion

In this study, in a public tertiary hospital, 28.4% of the parturients who received neuraxial analgesia for vaginal delivery progressed to cesarean. In similar studies, in parturients receiving neuraxial analgesia, the conversion rates to cesarean ranged from 21.4% to 28.4%.^11^, ^12^, ^13^ Cultural issues, patients’ demographic characteristics, and differences between public and private hospitals may be responsible for the differences between the incidences of cesarean rates in different studies. In the study by Bannister-Tyrrell and collaborators, reporting a 21.4% cesarean rate, epidural analgesia in labor was more common among women aged 35 years and older, primiparous women with labor induction, women at 41 weeks, and in private hospitals.^11^ Lawson and collaborators reported an incidence of cesarean rate of 28.4% after labor analgesia. They selected parturients who had not received augmentation of labor by oxytocin and those who received regional analgesia were more commonly nulliparous, obese, admitted to hospital as a private patient and had a higher socioeconomic status.^12^ Wang and collaborators recruited patients with cervical dilation larger than 1 cm and willing to have labor analgesia. Their mean age was 27 years, and gestational age ranged from 39 to 40 weeks. Analgesia was standardized as 15-mL epidural analgesic mixture in a single bolus of 0.125% ropivacaine with 0.3 μg.mL^-1^ sufentanil, followed by patient-controlled analgesic pump. The mean rate of cesarean was 23.0% and there was no difference according to initial cervical dilation.^13^ In Brazil, unlike in the past few years, currently, in public hospitals, parturients in labor can choose to undergo cesarean at any time, even when they are already under neuraxial analgesia for vaginal delivery. This fact was observed in 8.5% of our patients.

We found that older maternal age and longer duration of labor analgesia were associated with higher odds ratio of cesarean, whereas the administration of oxytocin after analgesia reduced the probability of a change in the type of delivery, in this case, more vaginal deliveries.

An observational study revealed an association between advanced maternal age and cesarean delivery by showing that primiparous women aged between 35 and 39 years had twice the risk of cesarean section, whereas women aged 40 years or older had this risk tripled.^5^ Studies have shown that cesarean rates are higher in women aged 35 years or older and that women over 40 years are at greater risk for emergency cesarean.^4^^,^^14^ This association occurs regardless of the administration of neuraxial analgesia during labor. Possible explanations for the increased chance of cesarean section with increasing maternal age may be the decline in physiological functions, such as those of the genital tract and its muscles, uterine muscle and hormonal system. The contractile function of uterine myometrial cells likely becomes less responsive to oxytocin or prostaglandins with aging, causing differences in the duration of labor.^15^

Regarding the increased risk of cesarean with prolonged time under analgesia, a positive relationship between analgesia and the extension of the first stage of labor has been observed.^16^ The interruption of painful stimuli reduces uterine activity due to decreased release of mediators involved in uterine contractility, such as prostaglandin F2α and oxytocin, resulting in a slowed labor progression.^17^ However, neuraxial analgesia reduces plasma adrenaline levels, which may favor uterine contractility and potentially shorten the duration of labor.^18^

Neuraxial analgesia may also prolong the second stage of labor.^11^^,^^19^ There is decreased effectiveness of maternal expulsive efforts due to abdominal muscle relaxation and reduced coordination of efforts during uterine contractions, in addition to pelvic diaphragm relaxation, which impairs fetal head rotation and engagement.^20^ Cheng and collaborators observed that patients under neuraxial analgesia had a one-hour longer duration of the second stage of labor than did those undergoing labor without analgesia.^21^ Lawson and collaborators reported that the mean duration of the second stage of labor was approximately 30 minutes longer with neuraxial analgesia, in addition to a twofold-increased risk in cesarean delivery.^12^

Neuraxial analgesia during labor may result in a significant extension of both the dilation and fetal expulsion phases. However, some studies have failed to associate longer analgesia duration with a corresponding increase in cesarean rates,^22^^,^^23^ which contrasts with the findings of our study.

Oxytocin is used to induce labor, augment uterine contractility, and manage labor progression. However, it can be associated with an elevated risk of instrumental vaginal delivery and cesarean.^24^ In patients in prolonged labor, oxytocin can shorten the time of labor by approximately two hours without significantly increasing the risk of cesarean or neonatal morbidity.^25^^,^^26^ A positive association between labor analgesia and oxytocin administration is observed.^27^ This may be attributed to the fact that epidural analgesia prolongs labor, especially the second stage, leading to the use of oxytocin to intensify uterine contractions and promote labor progression. Furthermore, neuraxial analgesia reduces plasma levels of endogenous oxytocin, justifying the more frequent use of exogenous oxytocin for labor induction.

In the present study, not using oxytocin during labor analgesia was the factor with the highest statistical weight in the predictive final model for cesarean. However, our data diverge from previous studies, which did not show any differences in cesarean rates when oxytocin was used for augmentation of spontaneous labor.^28^

The final model constructed with three predictor variables demonstrated a moderate predictive performance for unplanned cesarean delivery in parturients receiving analgesia for vaginal delivery, with an AUC of 71.8%. The construction of the predictive model involved progressive steps to select significant predictive variables (*step-up* procedure), based on the idea that a parsimonious model, i.e., with fewer variables, is preferable to a more complex one. An AUC with values between 70% and 90% is considered moderate with respect to its discriminatory ability, whereas values above 90% would indicate high predictive ability.^29^

The predominant reasons for cesarean are labor dystocia, which includes failure in cervical dilation and fetal descent, followed by changes in fetal heart rate, which may indicate fetal distress.^30^ The main causes of cesarean delivery that we observed were arrest of descent and fetal distress. Our findings agree with those in the literature, which identified arrest of descent as the most common indication for cesarean, both for women who received and for those who did not receive epidural analgesia.^2^^,^^15^^,^^19^

Our model can predict with moderate accuracy the occurrence of an unplanned cesarean in patients expecting to have a vaginal delivery under neuraxial labor analgesia. It is the first predictive model involving parturients receiving labor analgesia and may help doctors on changing treatments, specifically according to the variables of our model, to consider using oxytocin in older parturients in prolonged time under analgesia. Nonetheless, the model still needs external validation in different populations.

This study has some limitations. It is a single-center study conducted in a public hospital, limiting its generalizability to other types of maternity care units. There may also be other unmeasured confounders as we did not investigate aspects related to labor management, such as the frequency of pelvic exams, the analysis of each stage of labor, the dose of oxytocin administered, and the quality of uterine contractions after neuraxial analgesia. These factors could also help clarify some of the discrepancies found when comparing our results with those of other studies. However, we present a parsimonious predictive model contributing to a better understanding of the magnitude of factors associated with unplanned cesarean delivery in parturients under labor analgesia for vaginal delivery. Although the 70%‒30% split of the limited-size, single-center dataset may have influenced the final model, it achieved good performance metrics. Although we performed techniques to support the internal validation of the model such as train-test split, Hosmer-Lemeshow goodness-of-fit test, bootstrapping, and LASSO, the *step-up* approach might have led to an overfitting of the model, as suggested by the calibration plot. Nonetheless, as suggested by the decision curve, the model offers potential clinical utility in a fair range of threshold probabilities for identifying parturients who may require cesarean delivery. This study represents only the first of several steps for developing a practical tool to support medical decision-making. Future studies should test and validate the proposed model in multiple medical centers before it becomes available for clinical use.

## Conclusion

Among parturients undergoing labor neuraxial analgesia for vaginal delivery, 28.4% had unplanned cesareans. The set of variables, older age, longer time under analgesia, and not using oxytocin during analgesia increased the odds of a cesarean delivery, with moderate predictive value. The most common causes for cesarean delivery in these patients were arrest of descent and fetal distress. This is a preliminary study and, although the model shows moderate discriminative ability, further prospective external validation is needed before clinical application.

## Authors’ contributions

Paula Daniele Lopes da Costa: Conceptualization, investigation, formal analysis, methodology, visualization, writing - original draft, writing - review & editing; Murilo Henrique da Veiga Ferreira: Investigation, writing - original draft; Joelcio Francisco Abbade: Formal analysis, resources, writing - original draft; Claudia Garcia Magalhães: Formal analysis, resources, writing - original draft; Norma Sueli Pinheiro Módolo: Formal analysis, writing - original draft; Guilherme Antonio Moreira de Barros: Formal analysis, writing - original draft, writing - review & editing; Gabriel Ricardo Correa Turco: Investigation, formal analysis; Pedro Henrique Esteves Trindade: Data curation, formal analysis, methodology - statistics, writing - original draft; Paulo do Nascimento Junior: Conceptualization, formal analysis, investigation, methodology, project administration, supervision, validation, visualization, writing - original draft, writing - review & editing.

## Data availability statement

The datasets generated and/or analyzed during the current study are available from the corresponding author upon reasonable request.

## AI assistance disclosure

**Writing and English review**

During the preparation of this work, the authors used Rubriq/American Journal Experts (https://www.aje.com/rubriq) for English language editing. After using this tool/service, the authors reviewed and edited the content as needed and take full responsibility for the content of the publication. No other AI tool was used in the preparation of the manuscript.

## Ethics

This study was approved by the Institutional Ethics Committee (Plataforma Brasil, CAAE: 55925621.8.0000.5411; Research Ethics Committee, #5.278.695, issued on March 8, 2022, https://plataformabrasil.saude.gov.br/login.jsf;jsessionid=TSTL-1Zil8DbrAlv0WrlZXP9; https://www.fmb.unesp.br/#!/cep).

## Funding

Gabriel Ricardo Correa Turco received a scholarship from São Paulo State University, President’s office, PROPe, #5859/2022.

This research did not receive any specific grant from funding agencies in the public, commercial, or non-profit sectors.

## Conflicts of interest

The authors declare no conflicts of interest.

## Appendix Supplementary materials

Image, application 1

## Footnotes

Supplementary material associated with this article can be found in the online version at doi:10.1016/j.bjane.2026.844732.
