# Supplementary material for: Developing a concise multivariable predictive model for cesarean delivery following neuraxial analgesia during labor: a prospective observational cohort study
Source: Braz J Anesthesiol. 2026 Jan 25;76(3):844732. doi: 10.1016/j.bjane.2026.844732 (PMC12907217; doi:10.1016/j.bjane.2026.844732)

**BJAN-D-25-00282**

**Supplementary Material**

**Developing a concise multivariable predictive model for cesarean delivery following neuraxial analgesia during labor: a prospective observational cohort study**

**Table 1S** Findings of the Least Absolute Shrinkage and Selection Operator (LASSO) applied to the predictive final model after the step-up procedure using the training database.

| **Parameters** | **Estimated value** |
| --- | --- |
| Linear coefficient (α) | -0.5781784064 |
| Slope coefficients (β) |  |
| Patient age | 0.0050632024 |
| Time under neuraxial analgesia | 0.0006779397 |
| Oxytocin use after analgesia | -0.4018429469 |
| Best lambda | 0.0009040561 |

The training database contains 70% of the randomly selected patients using a random stratification sampling by groups (166 [71.5%] cases of vaginal delivery and 66 [28.5%] of cesarean).

**Table 2S** Findings of expected posterior variance applied to the predictive final model after the step-up procedure using the training database.

| **Parameters** | **Estimated value** |
| --- | --- |
| Linear coefficient (α) | 16.97139 |
| Slope coefficients (β) |  |
| Patient age | 6.366505^-04^ |
| Time under neuraxial analgesia | 2.234332^-04^ |
| Oxytocin use after analgesia | 2.330285^-06^ |

The training database contains 70% of the randomly selected patients using a random stratification sampling by groups (166 [71.5%] cases of vaginal delivery and 66 [28.5%] of cesarean).

**Figure 1S** Calibration plot with testing database, containing 99 patients (71 [71.7%] cases of vaginal delivery and 28 [28.3%] of cesarean). Blue line with dark dots shows the observed proportion of positive outcomes (y-axis) versus the mean predicted probability (x-axis) in each bin (dark dots) of predicted probabilities. Red dashed line (45° line) represents predicted probabilities exactly matching observed proportions. Dark dots above the dashed red line indicate underestimation of the risk (cesarean) while the one below dashed red line indicates overestimation of the risk.


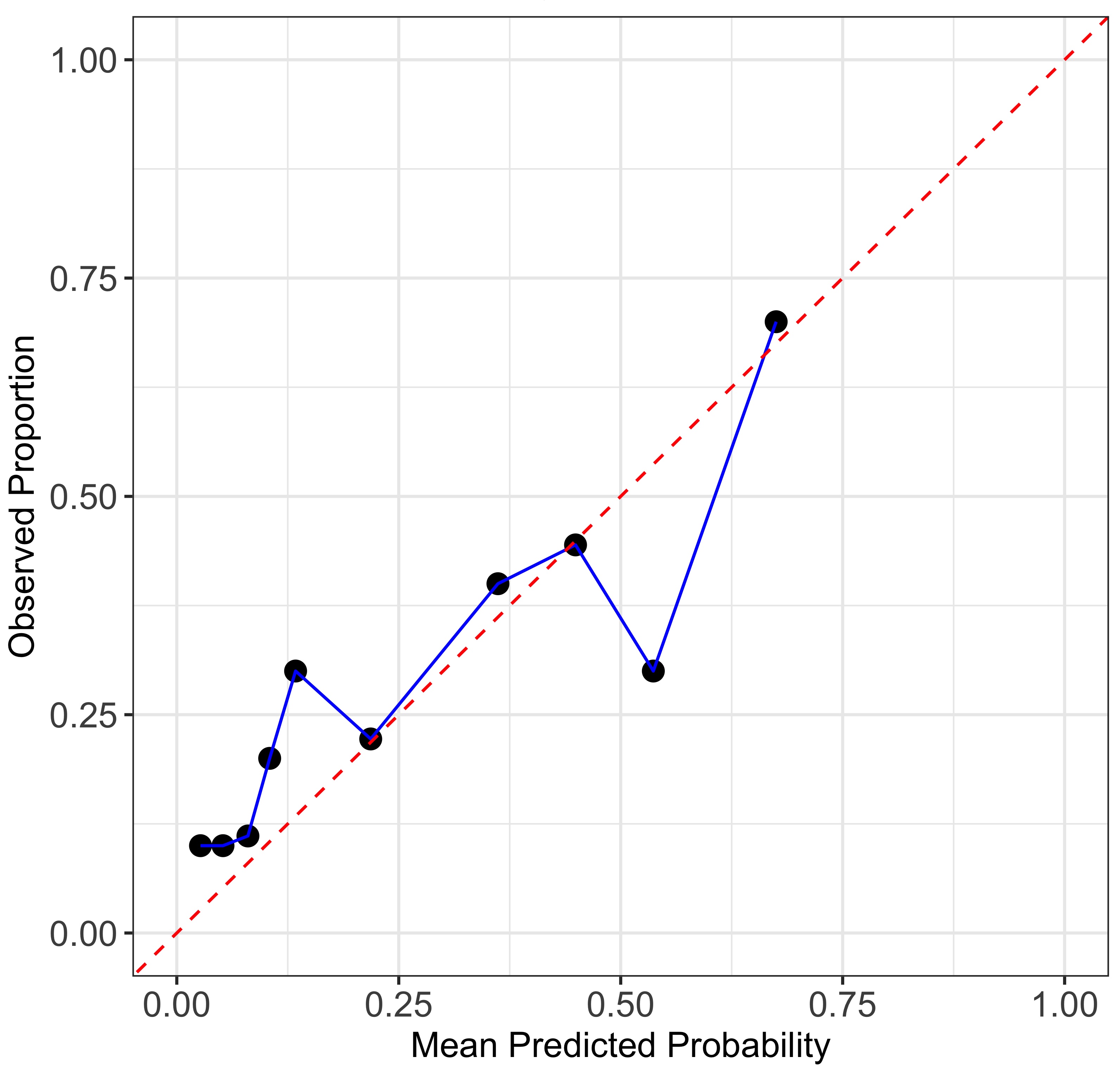


**Figure 2S** Decision curve analysis (using threshold probabilities ranging from 0.05 to 0.80, in increments of 0.005). The thick red line represents the threshold probabilities of the predictive final model, constructed from the testing database, and the respective net benefits. The thin red lines represent the lower and upper 95% Confidence Intervals. The grey thick line represents the net benefit of treating (cesarean) all patients. The grey thin lines are the lower and upper 95% Confidence Intervals. The black line, corresponding to zero in y-axis, represents the net benefit of treating (cesarean) no patients. There is benefit (e.g., higher number of true positives) when the red line is above both, grey and dark lines.


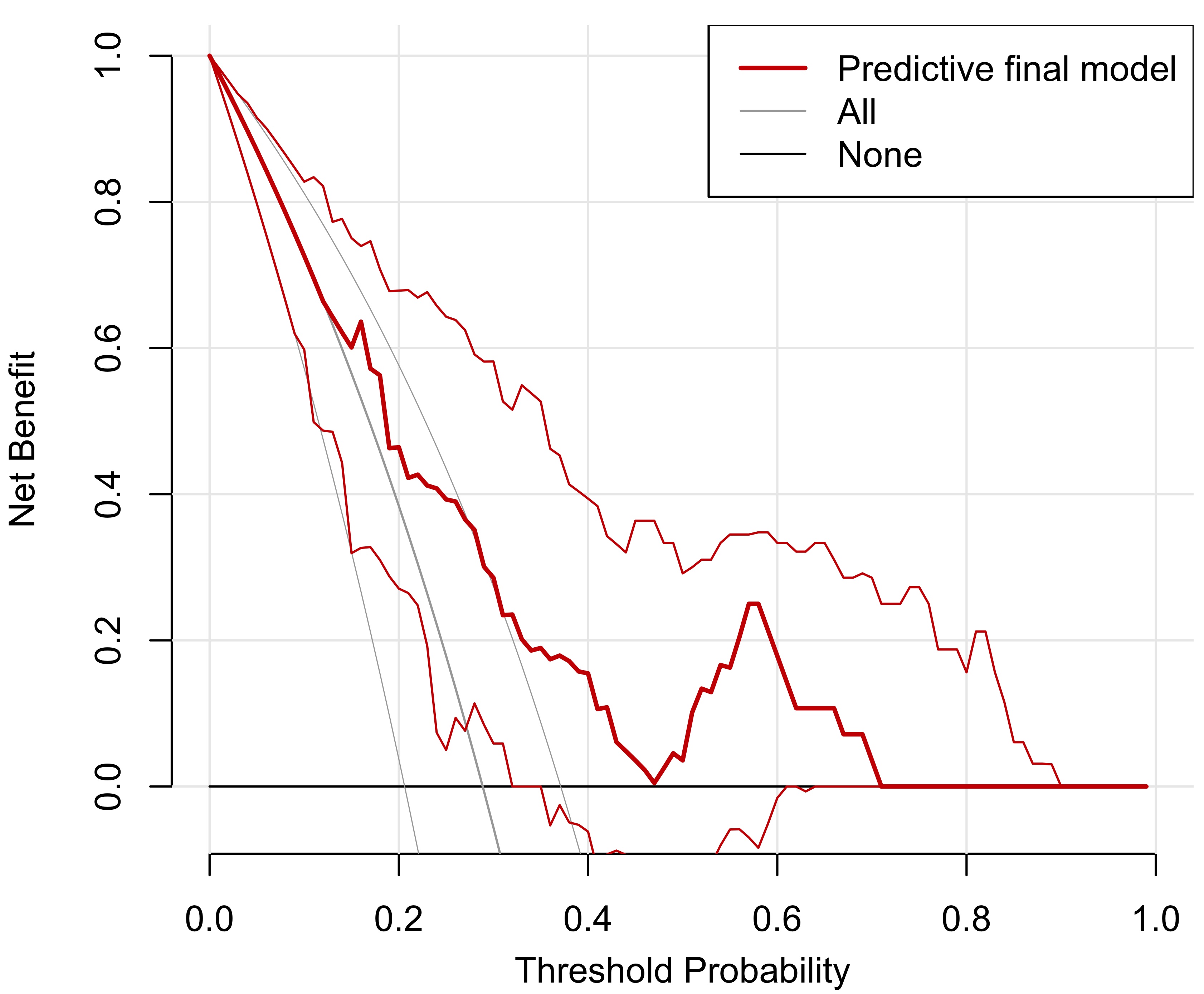

Supplement: Supplementary file 1 [file mmc1.docx]
